# The role of knowledge, risk perceptions, and cues to action among Iranian women concerning cervical cancer and screening: a qualitative exploration

**DOI:** 10.1186/s12889-020-09701-6

**Published:** 2020-11-11

**Authors:** Rahim Taghizadeh Asl, Liesbeth Van Osch, Nanne De Vries, Kazem Zendehdel, Mohsen Shams, Fatemeh Zarei, Hein De Vries

**Affiliations:** 1 Cancer Research Center, Tehran University of Medical Sciences, Imam Khomeini Hospital, Keshavarz Blvd, Tehran, Iran; 2 Department of Health Promotion, CAPHRI School for Public Health and Primary Care, Maastricht University, PO Box 616, 6200 MD Maastricht, The Netherlands; 3 School of Health, Yasuj University of Medical Sciences, Pasdaran 15 Blvd., Yasuj, Iran; 4 Department of Health Education and Health Promotion, Faculty of Medical Sciences, Tarbiat Modares University(TMU), P.O. Box: 14115-331, Tehran, Iran

**Keywords:** Cervical cancer (CC), Qualitative study, Perception, Awareness, Cues to action, I-change model

## Abstract

**Background:**

Iran has a low incidence but higher rate of death from cervical cancer (CC). The country is in the process of implementing an organized screening program including HPV testing and cytology. Studies show high dropout in continued testing among eligible women. This qualitative study aimed to explore women’s awareness regarding CC and CC testing and the role of knowledge, perceived risk, and cues to action in this process.

**Method:**

Through a qualitative study based on the Framework Method, we recruited 81 women aged 25–65 who participated in 15 focus group discussions (FGDs) and two in-depth interviews in Tehran. The interviewees were selected purposefully during January to May 2015 from households belonging to different socioeconomic classes until data saturation. The data were acquired through 11 open-ended questions and 32 related probe questions. All interviews were transcribed and independently analyzed by two researchers (Kappa and agreement testing respectively: 0.77, 97.11%).

**Results:**

The coded texts were categorized under three themes and 13 subthemes. The three thematic areas referred to knowledge, cues to action, and perceived risks regarding CC and screening. The results showed that women had limited and unspecified knowledge about CC and screening, compounded by misconceptions regarding infection and cancer prevention measures. Social and cultural barriers hindered proper communication between health system/providers and clients and within communities on subjects related to CC and screening. The perceived risk of getting CC was low because of overestimating the role of hereditary factors for CC, difficulty in differentiating between cancer and sexually transmitted infections (STI), and the absence of visible symptoms.

**Conclusion:**

The results indicate a strong need to invest more efforts to improve health education and communication in the current national health program to promote awareness of the need to screen for CC through, for example, establishing correct knowledge and risk perceptions among women. In addition, this intervention should address women’s social environment in order to prevent misconceptions being communicated to women.

**Supplementary information:**

**Supplementary information** accompanies this paper at 10.1186/s12889-020-09701-6.

## Background

CC is the fourth most common cancer among women worldwide with a total of some 570,000 new cases and 6.6% of all female cancers in 2018 [1–4]. About 86% of CC cases and almost 90% of deaths due to CC occur in low- and middle-income countries [1, 3]. High mortality from CC can be reduced significantly by comprehensive health programs for prevention, screening and early detection, effective treatment, and care [1, 5]. The epidemiology of CC in Iran, both through the pathology-based cancer registry (ASR 2.2 per 100,000) [2, 6] and the population-based registry (6 per 100,000, 2012) [2, 7], shows a low incidence but high mortality rate to reported incidence (54%) [2] meaning that CC is a health priority.

CC control guidelines, including those of WHO [7], the WHO package of essential non-communicable disease (NCD) interventions for primary health care (PHC) in low resource settings [8], and cohort studies in 13 European countries demonstrate that integrated screening sequence modalities including HPV testing, visual inspection with acetic acid (VIA) and cytology testing (Papanicolaou test; Pap smear) within an organized testing strategy combined with HPV vaccination for all girls by the age of 15, and universal access to treatment and care for identified cases is an effective and feasible strategy for eliminating CC [2, 4, 7, 9, 10], thus contributing to decreased cancer morbidity and mortality [11, 12]. In addition to the general phenomenon of changing sexual behavior and risk factors [12, 13], social and cultural challenges regarding sexual education [13, 14] point to the necessity of implementing well-organized screening programs including HPV testing and cytology [15–17].

By 2016, the Islamic Republic of Iran was implementing an opportunistic testing strategy (exclusively cytology-based and testing) for cervical cancer screening (CCS) every three to 5 years for all women aged 30 to 65, integrated within the existing PHC services [18]. There is no national surveillance system or comprehensive epidemiologic data for CCS in Iran. Based on the results of small surveys (e.g. 2009) in limited populations, the attendance rate for CCS was around 65% for attending at least once and less than 30% for regular attendance [19]. In late 2016, Iran launched the specific health intervention package of combating NCDs [20]. Consequently, the policy for CCS as part of the NCD strategies was modified to an organized testing strategy including HPV- and cytology-based testing with an estimated coverage rate in the pilot area of about 10% in 2017 [1]. According to an updated national protocol for CCS (not yet implemented at the countrywide level), all women aged between 30 and 59 are eligible for early diagnosis check-ups that include history-taking and a general examination/observation every 5 years. A sequence is performed of HPV-testing followed by Pap smear testing in cases where questionable signs are evident or every 10 years for asymptomatic women. General examinations will be applied for women after 50 but testing will be voluntary-based [21–23].

Several factors have been found to be associated with the low attendance of CCS in various countries. Some of these pertain to socio-demographic factors such as access to services and financial issues [24, 25], lack of advice from a health care provider (HCP) [19, 25, 26], and social and cultural barriers [24, 27, 28]. Others are related to women’s knowledge regarding the existence of HPV infection and CCS programs [26, 29–32], factors influencing the awareness of the need to be screened [33, 34], misconceptions about suseptibility to CC [34], and contextual cues such as cancer in the family [19]. Studies have also revealed that factors such as the attitude toward cancer [33], subjective norms [27] and self-efficacy [34, 35] are also associated with women’s attendance in CCS.

The newly established strategy in Iran needs some regulation for developing proper health communication and promotion interventions in order to improve and retain attendance in CCS. A first step is to create awareness among women in Iran concerning the availability and need for CCS and the efficacy of screening which appears to be important as studies showed a lack of this awareness [36, 37]. Awareness of CC and screening is a prerequisite for motivating attendance and is also acknowledged as an essential phase preceding motivation in behavior change models such as the Integrated-Change (I-Change) model [38] predicting health behaviors including participation in screening programs [34]. Hence, knowledge and risk perceptions may not always influence behavior directly but serve as distal factors influencing instance motivational factors such as attitudes and self-efficacy beliefs [39, 40]. According to the I-Change model, important determinants of awareness are: (a) having sufficient knowledge about CC and the possibilities for screening; (b) feeling at risk of getting CC; and (c) receiving sufficient cues that prompt a person to think about the need for CCS. In Iran, very limited information exists about these determinants of awareness of CCS [37] – information that is relevant to develop health communication interventions.

In conclusion, the rationale for conducting this study in the absence of a national surveillance system, pertains to limitations in the scope and generalizability of previous studies and the lack of comprehensiveness of reported associated factors needed for predicting behavior change. Consequently, the aim of this study is to explore knowledge, cues to actions, and perceived risks of women about CCS. As several studies suggest that screening attendance and its determinants may differ per educational level and socio-economic status [41, 42], we will also explore the potential differences related to these variables. The results from this study will be most interesting for low-epidemic Islamic countries with limited awareness of CC considering cultural, social, and religious sensitivities. In addition, these findings will be helpful for developing effective health promoting interventions for Muslim women from Islamic countries living in Western or non-Islamic countries as well in order to provide them culturally tailored information suitable to their background [43–45].

## Method

This qualitative study was based on the Framework Method [46] and was conducted using FGDs and in-depth interviews. We have used the four-dimension criteria [47] summarized in Table 1 and detailed subsequently to ensure and explain the trustworthiness of the study in all steps from design to implementation, analysis, and interpretations.

**Table 1:** Four Dimension Criteria to Assess the Rigor of this Qualitative Study

Rigor Criteria	Strategies applied in our study
Credibility	• 17 interviews, 60 min each, January to May 2015 (five months) • Interview protocol, tested in two pilot interviews • Skillful researchers for interview and data analysis • Two moderators for each interview, one as interviewer and one as note-taker • Immediate debriefing after interviews • Double checking transcribed interviews notes taken by both researchers
Dependability	• Prepared study protocol and briefing before interviews • Track record of interviews and detailed information related • Coding accuracy and inter coders’ reliability testing (Kappa agreement testing) • Using software (NVIVO10) for analysis
Confirmability	• Immediate descriptive review of transcripts to identify diversities and similarities of data
Transferability	• Sampling based on Urban HEART results for classifying districts and selecting interviewees from all socio-economic classes • Examining repeated coding after each interview and not arisen new nodes and additional subthemes in the final analysis

Through a deductive approach, a semi-structured interview guide was developed for this study based on the I-Change model [38] as the framework for understanding the awareness and motivation concerning participation in CCS programs. The interview guide was composed of 11 main open-ended questions and 32 related probe questions (for deepening the interview in case of incomprehensive answers by interviewees) (Additional file 1). We focused on knowledge about CC and Pap smear testing, perceived susceptibility and severity of CC, cues to action, advantages, and disadvantages of testing, as well as barriers and motivating factors, self-efficacy, and action plans. For this paper, we focused only on awareness factors (pre-motivational components) and related constructs including knowledge, perceived risks, and cues to action. The interview guide was reviewed by a group of experts and tested in two FGDs with eligible women (*N* = 12) and it was used in the study after finalization regarding method and language. The study has received ethical clearance by Tehran University of Medical Sciences (TUMS) (reference number 24890, 11/08/2014) and was registered in Iran Clinical Trials (IRCT2014092918120N1) [48].

### Setting

The study was carried out in Tehran, a metropolitan city with some eight million inhabitants. The Municipality of Tehran has categorized its 22 districts (zones) into five categories using the Urban Health Equity Assessment and Response Tool (Urban HEART) which takes health and social determinants [49]. A reference indicator for this classification was life expectancy at birth. We recruited our population study from randomly selected districts from all determined categories reference to the Urban HEART project, as a purposive sampling frame shown in Table 2.

**Table 2:** Grouping of Districts in Tehran Based on Life Expectancy

Life expectancy (years) in 2010	Districts code	Selected districts for FGDs
79.1 to 78.3	1, 4, 5	4 (3 FGDs)
78.2 to 77.4	2, 3, 22	3 (2 FGDs), 2 (1 FGD)
77.3 to 76.4	6, 15, 20, 21	15 (2 FGDs)
76.3 to 75.5	7, 8, 14, 18	8 (3 FGDs)
75.4 to 74.5	9, 10, 11, 12, 13, 16, 17, 19	13 (2 FGDs), 12 (2 FGDs)

### Recruitment of participants

A total of 15 FGDs and two in-depth interviews were conducted. The total number of within attending the interviews was 79 and two for in-depth interviews. All FGDs were conducted in nine public health care centers located in seven selected districts in Tehran; the centers were selected based on their availability and cooperation.

All women were invited to the interviews by the above centers located in the selected districts of the city (see Table 1). The inclusion criteria for the study pertained to residence in the selected zone, and being married, aged 25–65, and Farsi-speaking. The staff of the centers working in the reproductive health units contacted the women by phone (if accessible, in order from family health profiles) or directly (in order of their referring to the health center) to invite them to participate in the study. Due to the low proportion of women with higher education and employment among the interviewees, we decided to conduct one interview (FGD 17) at the workplace (for the convenience of the interviewees) and two in-depth interviews with employed women with higher education who were introduced by the staff center based on the researchers’ request.

### Data collection and analysis

RTA (MD, MPH) and SZ (PhD in health education), both with good experience in qualitative research design and implementation, moderated all interviews during January to May 2015. Two researchers conducted the interviews (always female and male together), one as the interviewer/moderator and one as the note taker.

First, the interviewers were introduced to interviewees by the staff of each center thereby providing an overview of the interview and its objectives. Subsequently, the process and ground rules of the interview (time, role of facilitator and interviewees, respect, and confidentiality) were explained and a consent form was read for all. The consent form clearly stated that the results would be published for scientific purposes with consideration for interviewees’ confidentiality and anonymity. The participants were informed that they could decide to stop and terminate the interview at any time without having to give an explanation. Before starting the interview, they were asked for their consent regarding audio recording of the whole interview. All the women who provided verbal consent continued to participate in the interview. Each interview comprised an introduction and collection of information on demographic characteristics (age, marital status, employment, literacy) and was completed in approximately 60 min.

After each interview, both the moderator and note taker reviewed the notes taken and consolidated them to be incorporated in the interview text when the audio file was transcribed. All audio files were transcribed by a typist and double-checked by the interview team. The transcribed data was descriptively reviewed to identify variation and similarity of information and expressions between FGDs while the interviews were in progress. All the names were coded in the interviews and the transcribed data was finally used for detailed analysis.

All the transcribed interviews were entered in the NVIVO 10 software and the two interviewers who were familiar with the interviews applied multiple-step analysis, starting with inductive coding of the data to identified nodes and classifying them in order to make the different parts of the data systematically comparable to each other. Ultimately, they completed the analysis by a deductive thematic categorization (framework-informed based on the I-Change model). Four randomly selected FGDs [4, 5, 9, 11] were used for extracting a unique list of nodes and comparison of coding by the two interviewers as analyzers. The results for Cohen’s Kappa and agreement testing showed 0.77 and 97.11%, respectively. Next, the researchers coded all interviews and classified all codes under specified subthemes and themes. Eventually, all themes were clustered in accordance with the I-Change model.

After completing 10 FGDs (two in each district), we performed a preliminary analysis on the data and then continued with one more interview in each area (including workspace) and two additional individual in-depth interviews. By analyzing them, we noticed redundancy in coding and no new nodes or themes arose which indicated data saturation. For analytic purposes, we reclassified the selected districts for interviews in four socioeconomic groups (SEGs) using socioeconomic position (SEP) scoring developed based on Urban HEART data [50] and we compared summarized expressions and coded texts from each SEG wherever possible.

## Results

### Characteristics of the sample

Table 3 shows a summary of the demographic characteristics of the interviewees. No illiteracy was reported and five out of 81 respondents (6%) reported having completed university education.

**Table 3:** Demographic Characteristics of the Participants in FGDs

Characteristics	Frequency	Percentage (%)
Age	Mean = 39.3 (SD = 10.8)	
25–34	32	38.6
35–44	26	31.3
45–54	14	16.9
> 54	9	10.8
SEG		
High	14	17.3
Upper-Middle	36	45.7
Lower-Middle	20	24.7
Low	10	12.3
Occupation		
Employed	5	6
Housewife	76	94

The results of the study focus on three main components of the pre-motivational stage of the I-Change model [38]: knowledge, cues to action, and perceived risks. Each component or theme contains subthemes accordingly which were summarized in Table 4 with the following details.

**Table 4:** Themes and Subthemes of Women’s Awareness of CC and CCS

Theme	Sub-theme
Implicit knowledge about CC and CCS	*● Lack of explicit knowledge on CC* *● Limited knowledge about signs and symptoms of CC* *● Lack of specific knowledge on CC testing, its frequency and timing* *● Deficit in knowledge about risk factors and causes of CC* *● Appropriate knowledge on conditions/ requirements for CC testing* *● Limited and unspecified knowledge on preventive measures, care and treatment concerning CC*
Inaccurate perceived risk of CC and CCS	*● Low perceived susceptibility to CC due to misconceptions* *● Aggravated severity due to social and cultural misperceptions*
Lack of perceived cues to action regarding CC	*● External cues:* *◦Limited interpersonal and public communications about CC due to cultural considerations* *◦HCP advice as powerful external prompt* *● Lack of internal cues:* *◦Socially imposed self-deprioritization, lack of symptoms and misinterpretation of symptoms*

### Implicit knowledge about CC and CCS

The results of the interviews revealed that women’s knowledge and information about CC and its signs and symptoms as well as CC testing was neither specific nor explicit. Tacit knowledge is embedded in practical experiences not achieved through systematic or formal trainings and not from standard documents and it is nonspecific. By contrast, explicit knowledge is evidence-based, specific, and precise knowledge which could be articulated and communicated systematically [51].

The results revealed different subthemes for this area which are discussed below.

#### Lack of explicit knowledge on CC

Most of the women had heard about CC in general but had not specifically mentioned the exact name of CC in the discussion. According to participants, older women have less knowledge than the younger generation. The majority of interviewees were also unable to differentiate between CC and other genital cancers (e.g. uterine cancer) and did not know the affected organ.

*“We don’t know anything about this (CC), we just know that a test is taken every six months or every year to see if a woman has uterine cancer or not.” (participant 1, > 54 years old)*

Half of the participants (almost equally distributed over the socioeconomic classes) referred to CC using nonspecific terms, mainly “women cancer” or “uterine cancer” without differentiating the uterine and cervix.

“*… among the general population, I don’t hear anyone say CC, it is called uterine cancer.” (participant 13, > 54 years old).*

Participants from low and lower-middle SEGs reported the least accurate and explicit knowledge about CC in general while inaccurate knowledge (content and frequency) was almost the same among all SEGs.

#### Limited knowledge about signs and symptoms

Most women did not recognize the signs and symptoms of CC specifically and explicitly. They did not differentiate between CC and STIs, nor their signs and symptoms.

*“I think this cancer is very silent and the one that is affected does not see any symptom and cannot identify the disease herself.” (participant 55, 45–54 years old).*

Several women stated hemorrhage (vaginal bleeding) and vaginal discharge (extensive and smelly) as hallmark symptoms.

*“… it starts from the inner uterus and extends to the abdomen. One of our relatives was affected and within six months, when in hospital, her uterus was removed but it was rooted in her abdomen … I guess the first symptom is severe bleeding.” (participant 4, 25–34 years old).*

Others, however, referred to a lack of pain or symptoms.

*“… cervical ulcer does not have any specific pain sometimes, no consequence and symptoms, and if any symptom exists it could be foul-smelling vaginal discharge, for example.” (participant 55, 45–54 years old).*

Occasionally interviewees mentioned other signs such as abdominal pain, genital burning, and irritation. A few women mentioned warts as a sign of cancer and some others disagreed.

*“In my opinion, (vaginal) irritation, itching, and abnormal and smelly discharge can be a sign of having CC.” (participant 67, 35–44 years old).*

Women in the upper and lower-middle SEGs expressed more accurate knowledge about signs and symptoms than the high and low SEGs. Inaccurately assigned STI-related signs and symptoms to CC were very frequent among all groups.

#### Lack of specific knowledge on testing purpose, frequency, and timing

The majority of the women knew about CC testing but their knowledge in most of the cases was nonspecific and inaccurate concerning the purpose and frequency (timing) of testing.

They did not differentiate between testing for CCS and testing for STIs.

The CCS test was referred to by different names: Pap smear, cancer testing, women testing. It was very frequently stated that this test (Pap smear testing) diagnoses STIs and cancer (not specifically CC).

*“… this test (Pap smear) in fact shows infections in the body of women or disease inside of the uterus.” (participant 61, 35–44 years old).*

Almost all women who had previously taken the test stated that the first time they had undergone testing was when they visited their HCP (physician, gynecologist, or midwife) for reproductive health reasons after marriage, during pregnancy, or after giving birth. In addition, most of the women stated that they performed the test whenever their HCP advised to do so.

*“… we don’t know more; we only heard the name (of the test for cancer) and we only perform tests which are advised here (in the public health center).” (participant 2, 25–34 years old).*

A few women were aware of the correct frequency of testing as recommended in the national program for CCS. They expressed broad knowledge about the timing and frequency of testing, including twice a year, annually, every three years, etc., and the majority named annual testing.

*“As far as I know, we should perform this test every year; if three consecutive tests are negative, then there is no need to do it annually anymore and we can do it every three years. When we have passed the reproductive age and after aging, it (testing) also needs to be done and likewise after surgery (hysterectomy) which I had, after extraction of my uterus. I mean when you don’t have ovaries or a uterus, this test still needs to be done.” (participant 55, 45–54 years old).*

In addition, interviewees mentioned different ages and circumstances as the starting time for testing, including after marriage, before pregnancy, after giving birth (delivery), after turning 30 years old, from age 20 to 60 years, and after the menopause. Several women indicated that women should be examined and tested for STIs after marriage.

*“… after marriage, some problems (STIs) happen … when a woman feels that her discharge smells or is discolored, she should consult a physician.” (participant 69, 25–34 years old).*

Most women believed that samples are taken from vaginal discharges and did not differentiate between CC and STIs regarding care and management. The majority of the interviewed women described the testing as taking samples on slides and a few of them talked about taking samples in liquid mediums. A few participants noted that there are several ways to diagnose CC and mentioned biopsy and diagnostic curettage as additional options. Only one interviewee specified HPV testing.

*“… I know that a sample (for CC testing) is taken from (vaginal) discharges of women and sent to the laboratory for examination …*. *If the result of the test is suspicious, then the test should be repeated and in the case of an infection, it is cured with pills and medicine. It is important for women to be tested every six months and sometimes every year.” (participant 4, 25–34 years old).*

While the high SEG had the least knowledge about testing in general, most women in all groups expressed inaccurate knowledge about the frequency, purpose of testing, and type of specimens taken and did not differentiate between STIs and cancer.

#### Deficit in knowledge about risk factors and causes

Most women wrongly mentioned CC as a consequence of STIs, more specifically prolonged/chronic infections, and also described family history as a main risk factor for CC. In addition, they listed other risk factors including multiple partnerships, ignoring personal hygiene (cleaning and washing the genitals, daily changing of underwear), unhealthy nutrition (e.g. high intake of fast food), air pollution, stress, and wearing tight underwear. Only two participants mentioned smoking tobacco and alcohol use as possible risk factors.

*“… an unmarried person may be affected by this disease (CC) because of hereditary reasons.” (participant 63, 25–34 years old).*

The majority of women mentioned chronic and untreated STIs as the underlying cause for CC.

Women very frequently used terms of hereditary and genetic (more than 48 times) causes, and infections (STIs in general, more than 85 times) as being a cause of CC. Almost none of the women differentiated between cleanliness and hygiene in their statements and expressed an implicit understanding of hygiene to control risks.

*“I do believe that CC and other women cancers (related to genital organs) are caused by simple infections which are caused by our negligence such as neglected personal hygiene. We should be concerned about our sexual relations a lot, also about our underwear which I think is highly important.” (participant 72, 25–34 years old).*

Awareness of CC risk factors was highest among the high SEG but, most importantly, inaccurate knowledge about the main cause of CC (believed to be heredity) and misconceptions about hygienic measures for self-protection were highly stressed by almost all women.

#### Appropriate knowledge on conditions/requirements for testing

The majority of women were aware of practical preparatory measures and recommended considerations required before attending CC testing. They stated practical terms and conditions for testing including sexual abstinence, avoiding the use of vaginal douche and vaginal treatments (gel and cream), and recommended scheduling testing when not menstruating.

*“In general, no (vaginal) discharge should exist (at the time of testing) to avoid incorrect results of the test and a week should have passed after menstruation. Additionally, sexual intercourse should not have taken place in the previous 24 hours (before testing) and (vaginal) ointments should not have been used during the previous 48 hours.” (participant 74, 25–34 years old).*

All groups excluding the high SEG elaborated in detail on the required preparedness before testing.

#### Limited and unspecified knowledge on preventive measures, care, and treatment

Almost all women indicated not knowing how to prevent CC; they indicated that they were simply advised to perform the test.

*“In fact, we don’t know what to do or not to do to avoid facing the problem (of getting CC). We do not know anything in this regard at all. We only know that a test is being taken every six months or every year to identify if uterine cancer exists or not.” (participant1, > 54 years old).*

Most women understood that CC testing was a way to detect STIs early (without mentioning HPV) and they believed that STI prevention was essential in CC prevention. A few participants mentioned condom use as a preventive method. Only two women mentioned the availability of a vaccine and its preventive role; both women were highly educated.

The majority of women considered CC as a treatable disease if diagnosed early by extraction of the uterus and chemo- or radiotherapy before metastasis to other organs.

*“I think it is curable if diagnosed at an early stage. Chemo- and radiotherapies exist and eventually treatment is available. In the past, they may not have been able to diagnose it but now I see people who had problems which have been solved.” (participant 27, 45–54 years old).*

Low and upper-middle SEGs demonstrated more correct knowledge of preventive measures than low and high SEGs; however, the frequent referral (> 95 times) to personal hygiene and cleanliness (in general terms) as a preventive measure was notable among all SEGs.

### Inaccurate perceived risk of CC and screening

In general, the proportion of women who frequently cited their lack of perceived risk were almost the same in all SEGs. There was an overall low perceived susceptibility and high perceived severity among participants regarding CC. Most women believed that CC was a curable disease. However, some fear was reported due to extreme social and cultural consequences of CC (called as life interruption) by some of the interviewees.

This theme was categorized under two subthemes which explain three main factors related to risk perception: misperception of cause, misconception about STIs and CC, and avoidance of negative thinking.

#### Low perceived susceptibility to CC due to misconceptions

Insufficient knowledge about the cause of CC resulted in misconceptions regarding vulnerability to CC. Overestimation of the role of heredity of CC was an important misconception among women; most women considered themselves not at risk because there was no (known) history of CC in their family. They specified insufficient hygiene as an important risk factor for CC and perceived an association between STIs and CC. As a majority of women evaluated their personal hygiene measures as being sufficient to prevent infections, they did not consider their CC risk as being high. Those measures (e.g. changing underwear, washing themselves after sexual intercourse, not using toilet paper) pertain to cleanliness rather than specific hygienic measures.

*“I don’t think (I will get CC) because I observe hygiene always and we didn’t have such a thing (cancer) in our family.” (participant 40, 45–54 years old).*

A common social belief in Iran says that when you think of something, it eventually happens to you. This scares people away from exploring their concerns such as cancer.

*“Thinking about (CC) is very bad. I don’t like to think about it … When you are exploring more (about a disease), it seems that the disease grips you more. It (testing) is a useless cost and I should not consider it (CC) as important anymore.” (participant 27, 45–54 years old).*

#### Aggravated severity due to social and cultural misperceptions

Most women reported a high perceived severity of CC; severe consequences also included disruption of family life and social image.

*“… (women are) concerned that when it is known that they have CC, their life will be disrupted.” (participant 58, 35–44 years old).*

### Lack of perceived cues to action regarding CC

Most women reported few cues to action from their environment also due to an existing stigma on CC in their community and discrimination against women. Two different types of cues were mentioned: external cues and internal cues.

#### External cues

##### Limited interpersonal and public communications about CC due to cultural considerations

All women strongly emphasized the need for health communication and education on CC and CC testing. Most women indicated that talking about issues related to the sexual organs including CC and testing is stigmatized according to cultural and social norms.

*“CC is not an issue which you expect an affected woman to talk about with her friends and relatives and to open up about it. Usually she keeps it secret like breast cancer; she does not like others to know about her disease. For this reason, we don’t know how many of our relatives are affected, there is no communication about it, and we know cancer in general but not specifically.” (participant10, 25–34 years old).*

In comparison to other groups, the low SEG indicated a lack of communication about CC more frequently as a barrier to awareness. Different information sources were listed including HCPs, television, peers (relatives and volunteers), and others (internet, publications). Face-to-face counseling by public health services and HCPs including physicians, midwives, and others was mostly preferred by women as these consultations were mentioned as being transparent and provided the opportunity to pose questions. The lack of interaction (i.e. posing questions and receiving answers) was regarded as the main disadvantage of health education through the public media (e.g. television).

*“The public health center is credible (for health education); when I am in such a center, I feel comfortable because I can ask my questions from the doctors there but in that case (TV education), I cannot ask questions from anybody.” (participant 47, 35–44 years old).*

Women indicated being confident about having comprehensive knowledge about HCPs (particularly doctors) and trusting them. They were concerned that senior professionals spend less time with patients for health education.

*“Doctors do not open the discussion and don’t have time … when you ask more questions, doctors get upset.” (participant 36, 45–54 years old).*

A need for health education was commonly expressed among all SEGs with a higher frequency among high and upper-middle SEGs with the abovementioned preference scheme.

##### HCP advice as powerful external prompt

Being advised to go for screening by HCPs appears to be a very important cue encouraging attendance. Most of the women stated that they only participated in tests when their HCP advised them to do so; otherwise, they would not go for tests unless they had symptoms that might point to an STI.

*“Whenever my doctor advises me to go for a test, I go. Otherwise I would never go by myself, unless I had complaints.” (participant 39, 45–54 years old).*

The advice of an HCP was mentioned more often as the main motivation for testing among women in the upper-middle SEG than among women in the other SEGs.

#### Lack of internal cues

##### Socially imposed self-deprioritization and misinterpretation of symptoms

There was a general lack of perceived cues to initiate CC testing (apart from HCP advice); for most women, CC testing and more generally personal health care was not a main priority. Many women indicated that Iranian women prioritize family and household as a social value and criticize caring about personal health. Limited awareness of symptoms of CC is causing a misinterpretation of being healthy in the absence of visible symptoms, and neglecting routine examination and testing as follow-up.

*“Usually the last thing Iranian women have on their mind is (taking care of) themselves and their priority is thinking about other issues.” (Participant 1, > 54 years old).*

All groups very frequently expressed discrimination against women, self-deprioritization and misinterpretations of symptoms as barriers to participation in CC testing, with a slight dominance among the high SEG.

## Discussion

The aim of this study was to explore relevant factors determining women’s awareness of CC, awareness of CCS programs, and their perceived need to participate. The findings were summarized under three main themes including knowledge, perceived risk, and perceived cues to act.

Firstly, with regard to knowledge, our results revealed that a vast majority of women had general knowledge about CC and screening, but their information was implicit rather than explicit. They did not differentiate between CC and other genitourinary cancers, mainly uterine cancer. Most of the women did not explicitly name CC and they did not anatomically distinguish the cervix from the uterus. In addition, women did not specifically differentiate CC from STIs which was demonstrated by a lack of knowledge about signs and symptoms and causes and the relation between STIs, cancer, and CC testing. Differences between STI and CC management measures and interventions were not clear for women. Other national and international studies also reported a lack of sufficient knowledge about CC, a gap in identifying risk factors and causes of cancer, a lack of recognizing the cervix, and differentiating between STIs and CC [27, 36, 52]. In addition to the comprehensiveness of data and corroborating previous findings regarding women’s knowledge of CC and CC testing, the current study sheds light on the importance of establishing explicit knowledge and the role of detailed health education. Our results also showed that women’s knowledge about timing and regulations for CC testing was inaccurate and implicit and mainly reflected their experiences rather than accurate knowledge. Lack of knowledge and unconfident answers by women regarding timing, frequency, and procedures was also reported in other studies [33, 53]. However, women had detailed, accurate, and practical knowledge about the terms and conditions for attending CC tests which was not previously explored in other studies. This may reveal women’s attention for and interest in personal health issues and a potential capacity for learning from instructions and demonstrates a shortfall in the health system lacking systematic health education and uniform practices [52].

Secondly, our results depicted that many women held inaccurate risk perceptions concerning CC. The women in our sample overestimated the role of heredity, similar to the findings of other studies in Iran [27, 36] and other countries [53–55]; this is challenging since the role of genetic causes of CC is still not clear [56]. The vast majority of women reported low perceived risk and susceptibility regarding CC, again similar to findings also reported in other studies [33, 34, 37, 53]. We concluded that women regarded themselves at risk concerning STIs; however, they had poor understanding of the purpose of CCS. In concurrence with other studies, our respondents considered CCS as an intervention for STI management [27, 57–59]. A misconception was found concerning the role of cleanliness and hygiene to prevent STIs (HPV infection specifically) and CC; similar findings have been reported by other studies [33, 53]. However, the absence of STI symptoms did not make women feel the need to go for screening.

Thirdly, with regard to cues to action, our respondents disliked discussing and thinking about CC and their existing symptoms due to local beliefs about the consequences of such thinking and discussions, again a finding reported in other studies [24, 27, 53, 60]. Consequently, receiving a cue to act as a result of discussions with others is rare in Iran. HCP advice on performing a CC test was found to be a crucial factor – if not the most crucial – for undergoing screening. Women emphasized that despite all existing barriers and lack of awareness regarding CC and testing, they attended CC screening when their physician advised them to do so. Other studies also reported this positive influence [19, 61, 62].

Previous local studies showed a positive correlation between health literacy for CC and income level [63], a higher concentration of CC among the lower SEG in Tehran [64], and delayed diagnosis of CC among the same group [65]. Nevertheless, our findings did not indicate a constant pattern and do not suggest differences in awareness, knowledge, and risk perceptions between participants with different socioeconomic backgrounds. Health education and communication interventions are therefore suggested in all SEGs while further quantitative studies are recommended.

To the best of our knowledge, this was the first comprehensive qualitative population study on this subject in Tehran conducted with the participation of a large number of participants from diverse SEGs (spread over the city). Moderating interviews by two experts of both genders and the openness shown by the interviewees were other positive points of this study. The results of this study provide a wide range of comprehensive data on the aforementioned thematic areas and advocate the development of health communication interventions to promote adequate uptake of CC testing in Iran. However, the status ofsocio-cognitive factors related to CCS adherence should be further investigated to broaden understanding on this subject. In addition, quantitative studies are needed to identify statistical differences between groups and to identify which variables are most important for the realization of awareness of CC and CCS and to explore the association of individuals’ characteristics and identified factors with outcomes.

Although there is now a vaccine against CC, this is not yet available in Iran. Future preventive actions could entail making this vaccine available and to provide information about this: a strategy which is advocated by WHO suggesting to achieve a high coverage with both vaccine and a very sensitive screening test like a HPV test [17].

## Conclusion

The results of our study indicate low and implicit knowledge about CC and the preventive role of CC testing. In addition, women reported a low level of perceived susceptibility along with misconceptions influencing their uptake of preventive measures. Health communication campaigns, preferably using face-to-face methods in public health centers, should therefore stress correctness, explicitness of knowledge, and the importance of preventive measures and possible consequences of CCS ignorance and late detection. Campaigns should emphasize that a negative family history of CC is no guarantee for not getting CC as hereditary factors only play a minor role. HCPs and, more specifically, physicians were mentioned as trusted and preferred sources of education and their advice is one of the most influential factors for attendance indicating that they can play a vital role in the success of health communication campaigns for CCS in Iran.

## Supplementary information

**Additional file 1.** FGD interview guide.

## Data Availability

The datasets used and/or analyzed during the study will be made available by the corresponding author following a reasonable request.

## Abbreviations

CC: Cervical cancer
CCS: Cervical cancer screening
HPV: Human Papilloma virus
FGD: Focus group discussion
ASR: Age-standardized rate
WHO: World Health Organization
NCD: Non-communicable disease
PHC: Primary health care
VIA: Visual inspection with acetic acid
HCP: Health care provider
I-Change: Integrated-Change
TUMS: Tehran University of Medical Sciences
IRC: Iran clinical trial
Urban HEART: Urban Health Equity Assessment and Response Tool
SEP: Socioeconomic position
SEG: Socioeconomic group
STI: Sexually transmitted infection
