# Supplementary material for: The role of knowledge, risk perceptions, and cues to action among Iranian women concerning cervical cancer and screening: a qualitative exploration
Source: BMC Public Health. 2020 Nov 11;20:1688. doi: 10.1186/s12889-020-09701-6 (PMC7656771; doi:10.1186/s12889-020-09701-6)
Supplement: Supplementary file 1 — Additional file 1. FGD interview guide. [file 12889_2020_9701_MOESM1_ESM.docx]

Additional file 1: FGD Interview Guide

Cervical cancer screening Practice in Iran, interview with women in Tehran

1. What do you/women know about cervical cancer?
   1. Which organ is involved?
   2. What are specific relevant factors (preventing factors, risk factors, accelerating factors...)
   3. How is prevented and detected
   4. Treatability, curability and fatality
   5. Disability and affected functionality (personal, sexual…)
2. How you/women perceive yourself/themselves at risk of cervical cancer? Why?
3. What do you/women know about cervical cancer prevention program (pap smear testing)?
   1. Nature of test, what is the test? Why it is performed?
   2. How it is performed/ taken?
   3. Timing (in which period and frequency is taken)
   4. Importance (is it important and why?)
   5. Actions required (where it is taken? do you /women know where should go for test? by whom?)
4. How you and other women are/were made aware and from where (which sources) did you and other women get the information about cervical cancer screening?
   1. Source of information
   2. How you evaluate preference of women regarding source of information
   3. How you evaluate preference of women regarding type of education and awareness raising (methods and media, by whom, when, how)
   4. How do you evaluate influence and penetration of the sources
5. Which advantages do you perceive of participating (every three to five years) in cervical cancer screening? And how other women perceive advantages of participating in cervical cancer screening?
   1. Immediate/Short-term advantages and benefits
   2. Mid-term and long term (life-long)advantages and benefits
6. Which disadvantages do you perceive of participating (every three to five years) in cervical cancer screening? And how other women perceive disadvantages of participating in cervical cancer screening?
   1. Expected negative experiences ( in short-term or long term)
   2. Anticipated negative outcomes
7. What/Which factors are supporting/encouraging/ positive toward you/ women to attend in cervical cancer screening
   1. Personal factors / enabling factors/ assertiveness factors
   2. Other people (family, friends, peers…..)
   3. Social and environmental factors (social attitude and influence, insurance, financial issues, media/advertisement…)
   4. Health system (Health service and Health provider, Access)
8. What/which factors are against that/ discouraging/negative toward you and other women participate in cervical cancer screening?
   1. Personal factors / enabling factors/ assertiveness factors
   2. Other people (family, friends, peers,…..)
   3. Social and environmental factors (social attitude and influence, insurance, financial issues, media/advertisement,…)
   4. Health system (Health service and Health provider, Access)
9. Which situations/factors would make it easy/easier for you and other women to participate (start)/keep participating in cervical cancer screening?
   1. What to know about the test itself?
   2. Information and awareness about the test process and procedure?
10. Which situations/factors (would) make it difficult for you to participate (start)/ keep participating in cervical cancer screening?
    1. What to know about the test itself?
    2. Information and awareness about the test process and procedure? past experiences or stories/rumors
11. Which specific plans do you/women make to participate in cervical cancer screening?
    1. Prepare participation (preparatory plans)
    2. start or restart participation (action plans)
    3. Maintain participation (coping plans; how to overcome difficulties)
